# Dominant mid-to-high-frequency harmonics predict neural encoding and perception of concurrent vowels with large *f*0 differences

**DOI:** 10.1121/10.0038969

**Published:** 2025-08-01

**Authors:** Fan-Yin Cheng, Sarah Medina, Olaedochim Obinna, Spencer Smith

**Affiliations:** 1 Department of Speech, Language, and Hearing Sciences, University of Texas at Austin, Austin, Texas 78712, USA; 2 Department of Communication Sciences and Disorders, Montclair State University, Bloomfield, New Jersey 07003, USA, New Jersey 07003, USA

## Abstract

Concurrent vowel perception experiments have revealed the importance of fundamental frequency (*f*_0_) differences in speech stream segregation. Understanding neural processes that support speech streaming using *f*_0_ differences remains an active area of perceptual and neurocomputational modeling research. This study simultaneously measured subcortical neural encoding [frequency following responses (FFRs)] and cued vowel identification accuracy of 12 concurrent vowel mixtures with large *f*_0_ differences (>8 semitones) to assess whether *f*_0_-based neural channel selection predicted perception. Results indicated that the strength of *f*_0_-based neural channel selection for one vowel often depended on the identity of the competing vowel. FFR *f*_0_ amplitudes and cued identification accuracy were predicted by which vowel had more dominant spectral energy in the mid- to high-frequency range (>1500 Hz). Attending to the target vowel in each concurrent vowel mixture did not modulate neural representation of target or nontarget vowels. Together, these findings suggest that speech streaming using *f*_0_ differences may be supported by *f*_0_-based neural channel selection from dominant unresolved harmonics, at least for competing vowels with large *f*_0_ differences.

## I. INTRODUCTION

In real-world soundscapes, target speech often co-occurs with nontarget speech and other background noise. Despite this complex acoustic mixture reaching the ears, normal-hearing listeners are adept at perceptually segregating multiple sound sources (Bregman, 1994; Cherry, 1953; Darwin, 2005; Moore and Gockel, 2012). Various acoustic features have been shown to aid in perceptual segregation, including binaural cues (Darwin and Hukin, 1999; Gockel et al., 1999; Hartmann and Johnson, 1991; Kohlrausch et al., 2013; Sach and Bailey, 2004; Stainsby et al., 2011), onset and offset synchrony (Micheyl et al., 2010; Micheyl et al., 2013), and differences in fundamental frequency (*f*_0_; Arehart et al., 1997; Brokx and Nooteboom, 1982; Bregman, 1994; Flaherty et al., 2018; Flaherty et al., 2021). The latter feature has been studied extensively through concurrent vowel perception experiments (Arehart et al., 2005; Assmann and Summerfield, 1990; Culling and Darwin, 1993a,b, 1994; Summers and Leek, 1998; Chintanpalli and Heinz, 2013; Chintanpalli et al., 2016).

Concurrent vowel perception paradigms present listeners with simultaneous mixtures of two synthetic vowels. The duration and lateralization (e.g., monaural or diotic) of each vowel mixture are held constant, whereas the *f*_0_ of one speaker is parametrically manipulated. In most concurrent vowel experiments, the listener’s task is to identify each vowel, and the percent correct identification of both vowels is measured as a function of *f*_0_ difference (Arehart et al., 2005; Assmann and Summerfield, 1990, 1994; Culling and Darwin, 1993a,b; Summers and Leek, 1998; Chintanpalli and Heinz, 2013; Chintanpalli et al., 2016). Multiple studies have shown that as *f*_0_ differences increase up to ~1–2 semitones, perceptual identification of both vowels improves before plateauing (Assmann and Summerfield, 1990; Chalikia and Bregman, 1993; Culling and Darwin, 1993a,b; de Cheveigné et al., 1997a; de Cheveigné et al., 1997b). Interestingly, listeners require larger *f*_0_ differences, ≥4 semitones, to perceive that each vowel has a distinct voice pitch (Assmann and Paschall, 1998). These findings indicate that *f*_0_ difference is a salient cue in auditory segregation and relatively small differences can impart significant intelligibility benefits, even when both speakers are not perceived to have distinctly segregated voices.

“Place” and “place-time” computational models have attempted to explain concurrent vowel perception results (Assmann, 1996; Assmann and Summerfield, 1989, 1990; Chintanpalli and Heinz, 2013; Chintanpalli et al., 2014; Culling and Darwin, 1993a,b; de Cheveigné, 2023; Meddis and Hewitt, 1992; Prasad and Chintanpall, 2021; Settibhaktini and Chintanpalli, 2020; Smith et al., 2018). Both model types employ four basic processing stages in which (1) concurrent vowel stimuli are passed through an auditory filter bank; (2) the outputs of each filter are used to determine *f*_0_s through excitation patterns (place) or underlying periodicity at each place on the basilar membrane (place-time); (3) energy associated with each *f*_0_ is grouped to estimate spectral patterns of each vowel; and (4) estimated spectral patterns are used to classify both vowels in the mixture. Place models estimate the excitation pattern evoked by a concurrent vowel stimulus and, therefore, infer auditory nerve fiber rate-place activity along the length of the basilar membrane (Assmann and Summerfield, 1990; Scheffers, 1983a,b). These excitation responses are then passed through “harmonic sieves” or templates that extract equally spaced peaks from an excitation pattern to determine underlying *f*_0_s (Assmann and Summerfield, 1990; Duifhuis et al., 1982; Scheffers, 1983a,b). Neurophysiologic data from animals and humans offer some support for a place code for isolated and concurrent speech in the auditory periphery as well as the auditory cortex (Bendor and Wang, 2005; Fishman et al., 2013; Fishman et al., 2014; Fishman et al., 2016; Mesgarani et al., 2008; Qin et al., 2008; Walker et al., 2011). However, such a code may weaken at higher intensities in the majority of (high spontaneous rate) auditory nerve fibers (Assmann and Summerfield, 1990). Further, *f*_0_ extraction from excitation patterns is complicated when concurrent speech has similar *f*_0_s and individual harmonics are poorly resolved. In general, place models have *underestimated* human listener performance in concurrent vowel perception tasks (Assmann and Summerfield, 1990; Scheffers, 1983a,b).

Place-time models of *f*_0_ segregation may provide a more physiologically feasible alternative to place models (Assmann and Summerfield, 1990; Chintanpalli and Heinz, 2013; Meddis and Hewitt, 1992; Smith et al., 2018). These models derive *f*_0_s from concurrent vowel mixtures by capturing the dominant periodicity of auditory filter output waveforms or simulated auditory nerve fiber discharge patterns along the tonotopic axis. The rationale of this approach is based on the fact that the harmonic energy comprising concurrent vowels is concentrated around formant peaks and nonuniformly distributed along the basilar membrane. Thus, auditory filters with center frequencies near formant peaks of one vowel will preferentially phase lock to that vowel’s *f*_0_ (or low- to mid-frequency harmonics at the formant frequency) and vice versa; this phenomenon has been referred to as “channel selection” (Keilson et al., 1997). Channel selection has been observed in the behavior of the auditory nerve (e.g., Palmer, 1990, 1992) and brainstem neurons (e.g., Keilson et al., 1997). For example, cochlear nucleus chopper neurons phase lock to the *f*_0_ of one vowel in a concurrent vowel mixture when one of its formants occupies the neuron’s receptive field; the same neuron can be driven to phase lock to the opposite vowel’s *f*_0_ when one of its formants is shifted into the neuron’s receptive field (Keilson et al., 1997). Spectral shapes of concurrent vowels can be estimated with place-time models by grouping auditory filter outputs that oscillate at a common *f*_0_ periodicity and plotting autocorrelation strength as a function of auditory filter center frequency for each *f*_0_ group. Place-time models of *f*_0_ segregation using realistic neurophysiologic elements make quite accurate predictions of concurrent vowel perception as well as listener errors/confusions (Chintanpalli and Heinz, 2013); however, they cannot independently explain the observation that perceptual performance is above chance in cases where concurrent stimuli do not have *f*_0_ differences. Based on physiologic and modeling studies, it is likely that phase locking to specific formant frequencies aids listeners in the absence of *f*_0_ differences (Chintanpalli et al., 2014; Micheyl and Oxenham, 2010; Palmer, 1990; Young and Sachs, 1979).

Despite a large body of perceptual and computational modeling literature on concurrent vowel processing, little work has examined human neural responses to concurrent vowel stimuli. Most of this work has focused on cortical activity (Alain et al., 2005; Alain et al., 2017; Snyder and Alain, 2005). Alain et al. (2005) recorded cortical responses to concurrent vowels with variable *f*_0_ separations in passive and active listening conditions. In both conditions, the authors observed a negative-voltage wave superimposed on *N*1 and *P*2 components that grew in amplitude with larger *f*_0_ separations; they interpreted this finding to indicate pre-attentive cortical streaming based on *f*_0_ differences. An additional negative-voltage wave around 250 ms was also observed in active listening conditions, which was interpreted as reflecting effortful discrimination and classification following automatic streaming based on *f*_0_ differences. In a similar study, Bidelman and Yellamsetty (2017) found that the *P*2 component evoked by concurrent vowel stimuli in noise predicted perceptual segregation ability; however, they also reported that neural correlates of *f*_0_ discrimination may appear later (400–700 ms). In a follow-up study, the same authors observed that endogenous neural oscillations in the gamma frequency range predicted pre-attentive streaming of concurrent vowel mixtures and identification accuracy (Yellamsetty and Bidelman, 2018). Oscillations in the beta frequency range, which may reflect listening effort, predicted decision speed. These studies collectively demonstrate that pre- and post-attentive cortical mechanisms are involved in and, in some cases predictive of, concurrent vowel identification. However, because cortical evoked potentials mainly reflect “onset-type” and endogenous responses to auditory stimuli, they provide limited insight into the neurocomputational processes that extract important features (e.g., *f*_0_, formants) from concurrent vowels earlier in the auditory nervous system.

Subcortical auditory evoked potentials, such as the frequency following response (FFR), may be better suited to study neural encoding of concurrent vowel stimuli for multiple reasons. First, the FFR represents neural phase-locking to stimulus features (e.g., *f*_0_, formants) believed to be important to concurrent vowel identification (Chintanpalli et al., 2014). Yellamsetty and Bidelman (2019) were the first to demonstrate that FFRs could be evoked by concurrent vowel stimuli and that *f*_0_ and first formant information for each vowel could be extracted from FFR spectra. However, the spectral amplitudes of these components were not predictive of concurrent vowel identification accuracy, suggesting that the FFR reflects exogenous stimulus-based properties and may not be directly related to perceptual salience. Second, the FFR may be used to test specific tenets of computational models of concurrent vowel processing (e.g., *f*_0_-based channel selection, formant capture) in human listeners with some important caveats in mind. Whereas place-time computational models quantify dominant temporal periodicities from the outputs of *multiple* simulated auditory filter banks, the FFR represents pooled phase-locked responses of multiple subcortical nuclei to auditory filter outputs along the entire tonotopic axis, as measured from the scalp as a volume-conducted evoked potential. A critical finding from previous studies using complex tones or single vowel stimuli is that the amplitude of the FFR *f*_0_ component is significantly influenced by mid- to high-frequency areas of the cochlea in which *unresolved* harmonics interact and generate vibratory patterns at the *f*_0_ period (de Boer et al., 2020; Easwar et al., 2015; Easwar et al., 2018; Laroche et al., 2013; Zhu et al., 2013). Thus, consistent with channel selection data measured from single-unit animal recordings, FFR *f*_0_ amplitude may be an indirect measure of the relative power and distribution of formant energy between competing vowels, particularly in the unresolved frequency range. If FFRs capture *f*_0_ channel selection, an expected finding would be that the vowel in a concurrent vowel pair with dominant formant energy in the unresolved frequency range would evoke a larger FFR *f*_0_ component than the other less dominant vowel in the pair. Such a finding may provide a neural correlate to previous observations that one vowel in a concurrent vowel pair is often more perceptually dominant than the other, particularly as *f*_0_ differences increase (e.g., Chintanpalli and Heinz, 2013; McKeown, 1992; Yellamsetty and Bidelman, 2019). Further, an objective measure of channel selection to concurrent vowel stimuli may link human and computational data as well as provide a means to study auditory segregation early in the auditory system and the potential impacts of attention on this process.

This study measured FFRs to concurrent vowel stimuli during an *f*_0_-cued vowel identification task. We investigated how parametrically manipulating formant distributions (i.e., vowel identities) between concurrent vowels impacted neural *f*_0_ segregation. We hypothesized that neural channel selection would be more robust for vowels with dominant formant energy in the unresolved frequency range and perceptual identification of vowels with dominant mid- to high-frequency energy would be superior to their counterparts. We also hypothesized that neural *f*_0_ channel selection to a specific speaker would be enhanced when attention was directed to the vowel produced by that speaker based on previous literature demonstrating attention effects on subcortical neural processing (e.g., Cheng et al., 2021; Price and Bidelman, 2021).

## II. METHODS

### A. Participants

All study procedures were approved by the University of Texas at Austin Institutional Review Board (IRB), and all participants provided written consent prior to entering the study. The study sample consisted of 12 normal-hearing adults; all were American English native speakers (12 females, mean age ± 22.1 - years old, age range ± 18–31 years old). None of the participants had a history of neurologic or otologic pathology. All participants had normal air conduction hearing thresholds ≤25 dB hearing level (HL) bilaterally from 0.25–8 kHz.

### B. Stimuli

**FIG. 1.**
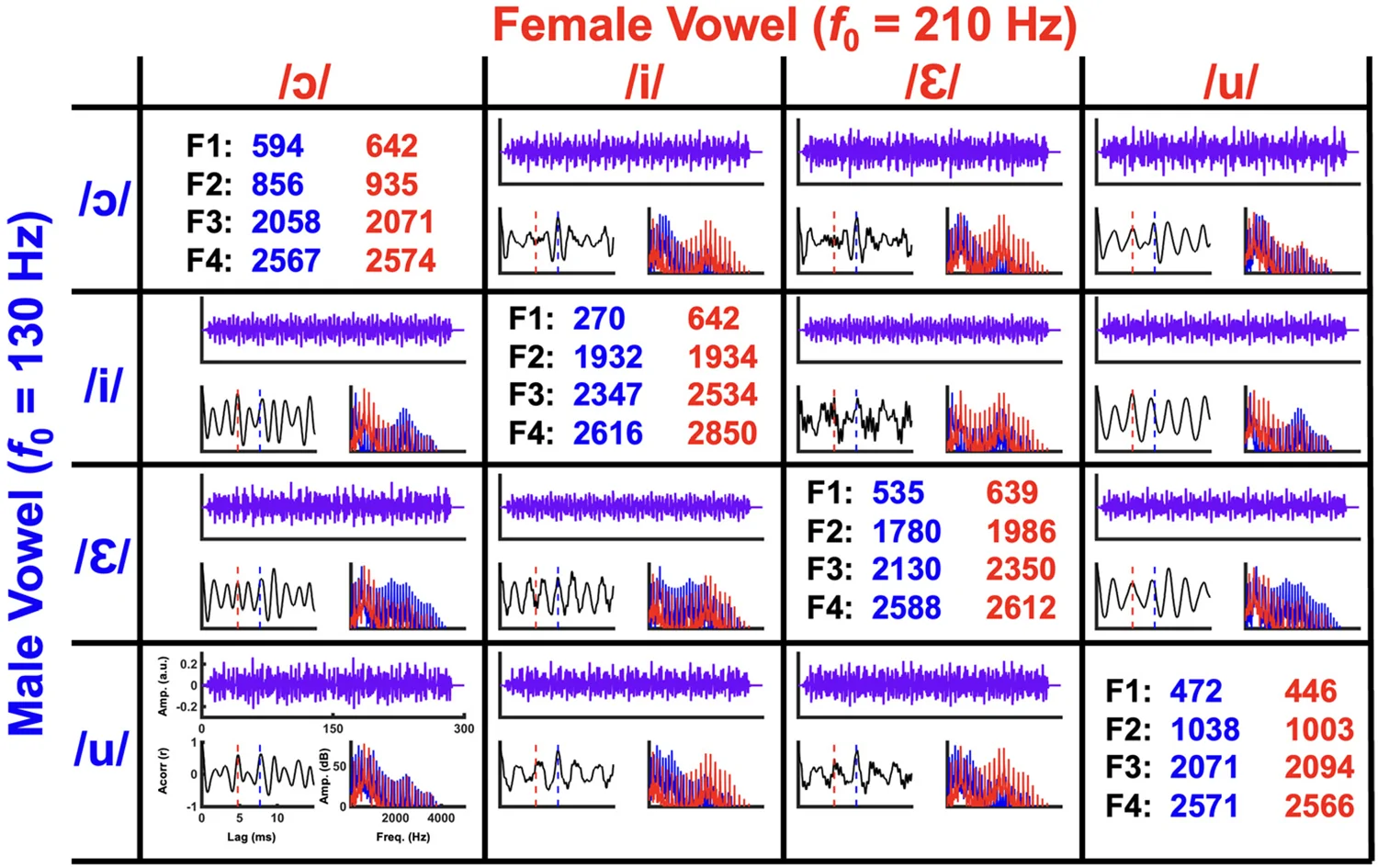
Concurrent vowel stimulus mixtures. Each concurrent vowel stimulus is represented in the matrix with male (blue) vowel identity denoted in the rows and female (red) vowel identity denoted in the columns. Purple waveforms at the top of each panel represent the time domain signal for each vowel mixture. Autocorrelation functions for each stimulus waveform are depicted in the lower left of each panel to identify dominant periodicities in the stimulus waveforms. The periods corresponding to male (7.7 ms) and female (4.8 ms) *f*_0_s are denoted with dashed lines. Spectra on the lower right of each panel depict color-coded contributions of male (blue) and female (red) talkers for each stimulus. Dual-vowel mixtures for identical vowels were not used in this study; instead, the panels corresponding to these omitted stimuli list formant frequencies (*F*1–*F*4) for male and female vowels /Ɔ/, /i/, /Ɛ/, and /u/, respectively.

Stimuli were created using combinations of four 250-ms-long synthetic vowels: /Ɔ/, /i/, /Ɛ/, /u/. Each vowel was generated using a Klatt Synthesizer in Praat software (Boersma and Weenink, 2024). Two versions of each vowel were created: one with an *f*_0_ of 130 Hz to represent a male talker and one with an *f*_0_ of 210 Hz to represent a female talker. These *f*_0_s have a larger semitone difference than most concurrent vowel stimuli (~8 semitones); however, they approximate average adult male and female *f*_0_s (e.g., Klatt and Klatt, 1990), which was critical for the *f*_0_-cued concurrent vowel task (described below). By permutating every possible combination of vowel pairs except for cases in which vowels were identical, 12 concurrent vowel mixtures containing a male and female talker were created (Fig. 1). The amplitudes of female vowels were adjusted in matlab (The Mathworks Inc., Nattick, MA) as needed to have equal loudness to male vowels; this adjustment was only necessary for female /i/ stimuli, which were attenuated by 2 dB sound pressure level (SPL). Each vowel mixture was presented through ER-3A insert earphones (Etymotic Research, Elk Grove Village, IL), and levels were calibrated to 75 dB SPL using a sound level meter (Quest, model 1800, Shoreview, MN) connected to a 2-cc coupler (GRAS, model RA0113, Holte, Denmark) with a 1-in. pressure microphone (GRAS, model 40EN).

**FIG. 2.**
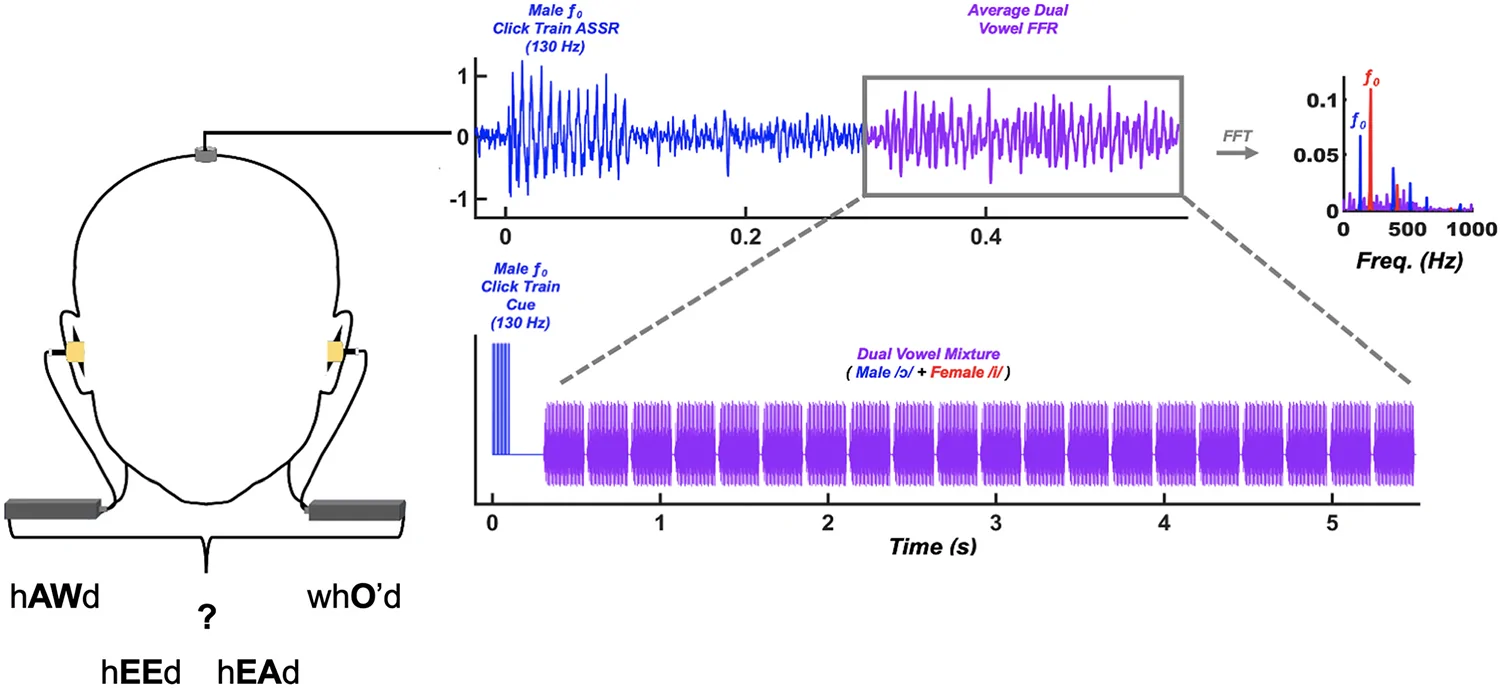
Clustered stimulus paradigm and FFR acquisition. A clustered stimulus example is depicted in the bottom panel. Each stimulus began with a 100-ms click train cue with either male (130 Hz, as depicted in this example) or female (210 Hz) *f*_0_ periodicity. After a 200 ms silent interval, a randomly selected concurrent vowel pair (male /Ɔ/ + female /i/, in this example) was presented 20 times in rapid succession (ISI = 10 ms). At the end of each stimulus presentation, listeners were prompted to select the vowel produced by the cued target (i.e., male or female) speaker using a response pad. The top panel depicts a typical averaged response to the clustered stimulus paradigm over 50 trials in a single subject. Note that the click train auditory steady state response (ASSR) waveform represents a 50-sweep average, whereas the FFR represents a 1000 sweep (50 trials × 20 presentations per trial) average. The denoised FFR spectrum with color-coded male and female components is represented in the upper right.

A clustered stimulus paradigm was used to expedite concurrent vowel FFR data collection (Bidelman, 2015). Each clustered stimulus began with a 100 ms click train with either 130 or 210 Hz periodicity (7.7 ms and 4.8 ms, respectively), followed by a 200 ms silent interval. The click train pitches matched the voice pitches of the target talker and were used to cue listeners which talker to attend to for the perceptual task described below. After the silent interval, 1 of the 12 concurrent vowel mixtures was randomly drawn and concatenated 20 times [10 ms induced spatial coherence (ISI); Fig. 2]. Thus, each trial was 5.5-s long. For each click train cue (*f*_0_ ± 130 Hz or 210), 50 repetitions of each clustered concurrent vowel stimulus were obtained.

### C. Procedure

The experiment was conducted in a soundproof, radio frequency (RF)-shielded booth with participants seated in a reclining chair. Clustered stimuli were presented binaurally through electromagnetically shielded ER-3A insert earphones, and visual prompts for the perceptual task were presented through a Dell personal computer (PC) monitor (Dell, Round Rock, TX). Stimulus presentation and response collection were controlled through the GenTask module in Neuroscan (Compumedics, Charlotte, NC) and presented in random order. Each stimulus cluster was presented 50 times, making the total presentation of each concurrent vowel mixture 1000 trials (20 repetitions/cluster × 50 clusters × 2 *f*_0_ cues).

#### 1. Perceptual task

For the perceptual task, participants were asked to identify only the vowel in each concurrent vowel mixture produced by the talker with the same *f*_0_ as the click train (130 or 210 Hz). After each stimulus cluster was presented, a visual display with four nonphonetic target vowels (“h**AW**d,” “h**EE**d,” “h**EA**d,” “wh**O**’d,” corresponding to /*Ɔ*/, /*i*/, /*Ɛ*/, and /*u*/, *respectively*) appeared on the PC monitor. Participants were prompted to identify the vowel as quickly as possible by pressing the corresponding number on a keypad (1, 2, 3, or 4). The next trial began 400 ms after a response was recorded. Note that this perceptual paradigm is more challenging than previous concurrent vowel studies, which typically prompted listeners to report *both* vowels that were heard, regardless of which speaker produced which vowel. We modified the classic paradigm to test whether attention enhances neural representation of the target vowel and/or suppresses the nontarget vowel. To perform above chance in our study, listeners had to perceptually segregate the cued speaker based on *f*_0_ cues and ignore the non-cued speaker. Prior to beginning the perceptual task, participants completed a short training in which they heard each isolated vowel presented five times in random order to ensure they could correctly identify it. Average perceptual accuracy for isolated male vowel tokens was 85% (SEM ± 63.2) and 82% (SEM ± 62.5) for females. The main purpose of this brief training session was to ensure that participants could perform the task for isolated vowels prior to the dual-vowel task.

#### 2. FFR acquisition and preprocessing

Electrophysiologic responses were collected with a Neuroscan SynAmps2 system (Compumedics, Charlotte, NC) and recorded at a 10 000 Hz sampling rate through a one-channel bipolar montage: Cz (+), forehead (ground), and linked *M*1/*M*2 (−). Continuous data were exported from Curry 8 software and analyzed offline in matlab (The Mathworks Inc., Nattick, MA). Continuous data were amplified (100 000×) and bandpass filtered from 70 to 2000 Hz. “Long” epochs were first extracted from −50 to 5900 ms (re: stimulus cluster onset) of the raw electroencephalogram (EEG). Long epochs were grouped by stimulus cluster type (2 speaker cues × 12 concurrent vowel mixtures), artifact rejected at ±75 *μ*V, and baseline corrected. To isolate FFR waveforms to *individual* concurrent vowel presentations, 20 “short” epochs (0–260 ms re: concurrent vowel onsets) were then extracted from each long epoch (Fig. 2). This procedure produced 1000 trials of each concurrent vowel mixture for each cue type (*M* or *F*).

Analyses focused on *f*_0_ amplitudes at 130 and 210 Hz obtained from FFR spectra. Formant frequencies were not assessed in this experiment because they were undetectable in many stimulus conditions. To minimize impacts of FFR residual noise levels caused by participant and task effects, we estimated the spectral noise floors for each participant, concurrent vowel mixture, and condition using a bootstrapping procedure (Zhu et al., 2013). For each subject, 400 single-trial FFR waveforms were randomly drawn from the 1000 cleaned short epochs for each condition. The fast Fourier transform (FFT) of each randomly drawn waveform was calculated. All 400 complex-valued spectra were averaged together to obtain a mean FFR spectrum. This procedure was repeated 100 times to generate distributions of raw spectral magnitudes for each frequency. Average spectra were calculated using the results of the 100 iterations described above. Spectral noise floors were estimated in a similar way. However, for randomly drawn single-trial FFR FFTs, phase components of the complex-valued spectra were randomly assigned values from 0 to 2P at each frequency. Randomizing phase in this way removes phase-coherent contributions from neural responses of interest and leaves participant-specific noise magnitude estimates across frequencies. The bootstrapped distributions of signal and noise spectra were analyzed using *F*-tests to determine if FFR components were present above the noise floors at 130 and 210 Hz (Cheng and Smith, 2022). Only FFR spectral amplitudes which showed significance above the spectral noise floor (*p* < 0.01) were kept for further analysis. For the remaining responses, “noise free” spectral amplitudes were obtained by subtracting the estimated noise floors (in *μ*V) from the FFR amplitudes at 130 and 210 Hz. Under this approach, 98% of FFR *f*_0_ spectral data were kept for analysis.

#### 3. FFR analysis

FFR *f*_0_ spectral amplitudes were initially analyzed using a linear mixed model (LMM) to assess acoustic and attention-based effects. The primary questions addressed by this LMM were (1) Do FFR *f*_0_ amplitudes differ by spectral frequency (130 vs 210 Hz)?; (2) do male and female speaker FFR *f*_0_ amplitudes independently differ by vowel identity (/Ɔ/, /i/, /Ɛ/, or /u/)?; (3) does FFR *f*_0_ amplitude for the *same* vowel change when paired with a different vowel?; and (4) do FFR *f*_0_ amplitudes differ based on the *f*_0_ cue (i.e., are FFR *f*_0_ amplitudes enhanced/suppressed based on which vowel is attended?). Independent variables included FFR spectral component frequency (130 or 210 Hz), vowel type (/Ɔ/, /i/, /Ɛ/, or /u/), vowel pair (/Ɔ/ + /i/, /Ɔ/ + /Ɛ/, /Ɔ/ + /u/, /i/ + /Ɔ/, /i/ + /Ɛ/, /i/ + /u/, /Ɛ/ + /Ɔ/, /Ɛ/ + /i/, /Ɛ/ + /u/, /u/ + /Ɔ/, /u/ + /i/, and /u/ + /Ɛ/; note that the first vowel denotes the male speaker and the second vowel denotes the female speaker), and click train cue frequency (130 vs 210 Hz). In the LMM framework, random effects were included to account for within-subject variability and repeated measures across trials. Fixed effects for FFR spectral component, vowel type, vowel pair, speaker cue, and their interaction were estimated. Pairwise comparisons of factor levels were conducted using model-based contrasts with Tukey correction to identify the sources of significant effects in R (R Core Team, 2024).

A second analysis examined whether spectral amplitude differences in the mid- to high-frequency range of concurrent vowel pair stimuli predicted FFR *f*_0_ amplitudes at 130 and 210 Hz. This question was based on the prediction that the vowel with dominant energy in the unresolved frequency range would evoke stronger phase locking at *f*_0_ than the less dominant vowel as a result of more robust channel selection of the dominant vowel. To obtain mid- to high-frequency dB differences between vowels, isolated vowel token wav files were first loaded into matlab and high-pass filtered > 1500 Hz using a tenth-order (60 dB/octave) Butterworth filter; this high-pass cutoff was motivated by previous reports that harmonics are unresolved approximately above the eighth to tenth harmonics in a complex, regardless of *f*_0_ (Bernstein and Oxenham, 2003). The normalized amplitudes of the high-pass filtered waveforms were converted to Pascals using an empirically calibrated scaling factor, and root mean square (RMS) pressure was calculated for each waveform. The RMS pressure of each high-pass filtered waveform was then used to calculate relative dB differences > 1500 Hz for all concurrent vowel combinations. In our approach, negative-valued spectral differences > 1500 Hz indicate greater spectral amplitude for the *female* speaker in the mid- to high-frequencies, whereas positive-valued spectral differences > 1500 Hz indicate greater spectral amplitudes for the *male* speaker. A second LMM assessed how spectral differences > 1500 Hz for each vowel pair were related to FFR *f*_0_ amplitudes.

#### 4. Behavioral data analysis

Vowel identification accuracy for each vowel pair and cue type was calculated by summing the correct responses and dividing by 50, the total trial number for each stimulus. Because there were four response choices per trial, chance accuracy was 25%. A LMM was used to examine the effects of spectral differences > 1500 Hz and speaker cue frequency on vowel identification accuracy. Fixed effects for spectral differences > 1500 Hz and speaker cue frequency as well as their interaction were estimated. Pairwise comparisons of factor levels were conducted using model-based contrasts to identify the sources of significant effects in *R* (R Core Team, 2024).

#### 5. Mediation analysis

If predictive relationships between spectral differences > 1500 Hz and male- and female-cued vowel accuracy were found, these observations may be mediated by the strength of subcortical neural phase locking to the target stimulus (i.e., channel selection), as indexed in the FFR. As described above, the strength of neural phase locking may be driven by the relative difference of spectral energy in the unresolved frequency range. A mediation analysis was conducted to measure relationships between spectral differences > 1500 Hz, FFR *f*_0_ amplitudes, and vowel identification accuracy. Before running the mediation analysis, we followed a three-step approach to assess the potential mediation effect of FFR *f*_0_ amplitudes (mediator) on the relationship between spectral differences > 1500 Hz (independent variable, *X*) and vowel identification accuracy (dependent variable, *Y*). First, we checked whether the spectral differences > 1500 Hz (*X*) directly affected identification accuracy (*Y*). Second, we examined whether the spectral differences > 1500 Hz (*X*) influenced FFR *f*_0_ amplitudes (*M*). Finally, we assessed whether the spectral differences > 1500 Hz (*X*) and FFR *f*_0_ amplitudes (*M*) influenced vowel identification accuracy (*Y*). If the relationship between *X* and *Y* is mediated by *M*, the direct effect of *X* on *Y* should weaken or disappear when the mediator (M) is included in the model (MacKinnon et al., 2007).

## III. RESULTS

### A. Stimulus and cue effects on FFR *f*_0_ amplitudes

#### 1. Stimulus *f*_0_ effects

**FIG. 3.**
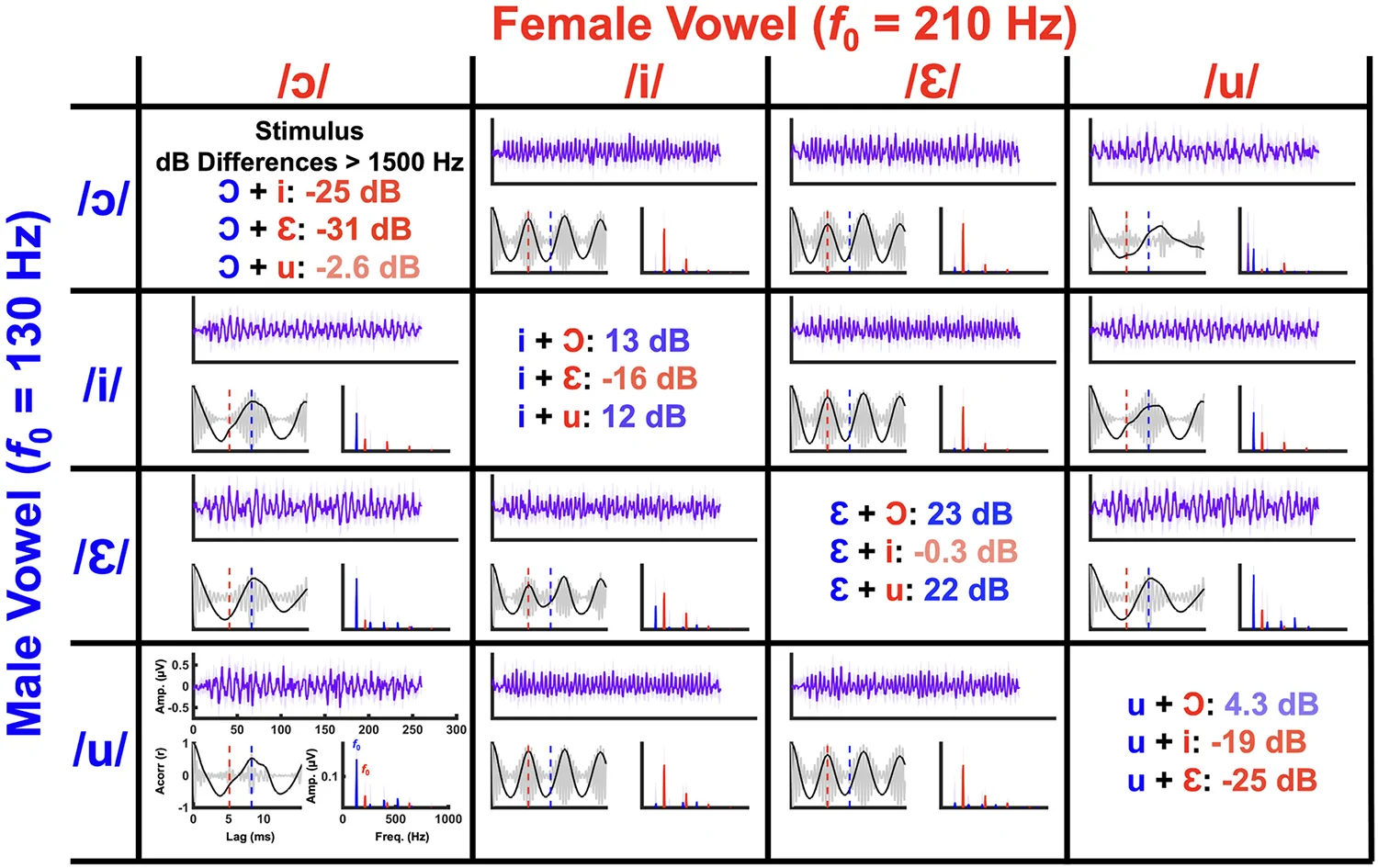
FFRs to concurrent vowel mixtures. Each concurrent vowel FFR is represented in the matrix with male (blue) vowel identity denoted in the rows and female (red) vowel identity denoted in the columns. Purple waveforms at the top of each panel represent the average time domain FFR for each vowel mixture. Autocorrelation functions for each average FFR waveform (black) are depicted in the lower left of each panel to identify dominant periodicities. The periods corresponding to male (7.7 ms) and female (4.8 ms) *f*_0_s are denoted with dashed lines. Autocorrelation functions for concurrent vowel stimulus waveforms filtered > 1500 Hz are also plotted (gray) for comparison. Average denoised FFR spectra on the lower right of each panel depict color-coded contributions of male (blue) and female (red) talkers for each FFR. Panels corresponding to the omitted same-vowel stimuli list dB differences > 1500 Hz between male and female vowels; note that negative numbers indicate greater female energy in the mid- to high-frequency range, whereas positive numbers indicate greater male energy. The dB difference values of each concurrent vowel stimulus are color-coded to indicate the magnitude of the difference > 1500 Hz (as in Fig. 4).

Average FFR waveforms and denoised spectra are depicted in Fig. 3. A significant negative effect of *f*_0_ frequency on FFR amplitudes was observed {*β* = −0.03, 95% CI [−0.04, −0.02], *t*(412) = −5.53, *p* < 0.001; standardized beta = −0.47, 95% CI [−0.64, −0.31]}. These results indicate that FFR *f*_0_ amplitudes were smaller for female vowels (210 Hz) compared to male vowels (130 Hz).

#### 2. Vowel identity effects

FFR *f*_0_ amplitudes at 130 Hz were significantly different for /Ɔ/ vs /i/ [b = 0.03, standard error (SE) = 0.01, *t*(177) = 2.84, *p* < 0.01], /i/ vs /Ɛ/ [*β* = −0.04, SE = 0.01, *t*(177) = −3.04, *p* < 0.01], and /i/ vs /u/ [*β* = −0.04, SE = 0.01, *t*(177) = −3.13, *p* < 0.01]. There was no significance for pairwise comparisons between other vowel types (all *p*-values > 0.05). FFR *f*_0_ amplitudes at 210 Hz were not significantly different between the four vowel types. These results indicate that there were baseline differences in FFR *f*_0_ amplitudes at 130 Hz across the four male vowels; the same was not observed for FFR *f*_0_ amplitudes at 210 Hz across the four female vowels.

#### 3. Vowel pair effects

FFR *f*_0_ amplitudes at 130 Hz were significantly different for /Ɔ/ + /Ɛ/ and /Ɔ/ + /u/ [*β* = −0.07, SE = 0.02, *t*(169) = 3.46, *p* < 0.05], /i/ + /Ɛ/ and /i/ + /u/ [*β* = −0.08, SE = 0.02, *t*(170) = −4.48, *p* < 0.001], /Ɛ/ + /Ɔ/ and /Ɛ/ + /i/ [*β* ± 0.08, SE = 0.01, *t*(169) = 6.56, *p* < 0.0001], and /u/ + /Ɔ/ and /u/ + /i/ [*β* = 0.1, SE = 0.01, *t*(170) = 6.54, *p* < 0.0001]. There was no significance for pairwise comparisons between other vowel pairs (all *p*-values > 0.05). FFR *f*_0_ amplitudes at 210 Hz showed a nonsignificant trend for /Ɔ/ + /u/ and /i/ + /u/ [*β* = −0.03, SE = 0.01, *t*(202) = −3.21, *p* ± 0.06]. Other vowel pair combinations showed no statistically significant differences. These results indicate that FFR *f*_0_ amplitudes for the *same* vowel were often impacted by the identity of the other vowel in the pair. This observation is represented in row- and column-wise *f*_0_ comparisons as a function of vowel pairing in Fig. 3.

#### 4. Speaker cue frequency

The effect of speaker cue frequency on the 130 Hz FFR *f*_0_ amplitude was nonsignificant (*β* = 0.001, *p* = 0.87). Similarly, the effect of the speaker cue on the 210 Hz FFR *f*_0_ amplitude was nonsignificant (*β* = −0.001, *p* ± 0.43). Together, these results indicate that cued attention did not alter FFR *f*_0_ amplitudes through enhancement or suppression of subcortical evoked neural activity. Consequently, FFR *f*_0_ amplitude analyses reported hereafter are collapsed across speaker cue frequency.

### B. Vowel pair spectral differences > 1500 Hz, FFR *f*_0_ amplitudes, and identification accuracy

#### 1. Relationships between concurrent vowel spectral differences > 1500 Hz and FFR *f*_0_

**FIG. 4.**
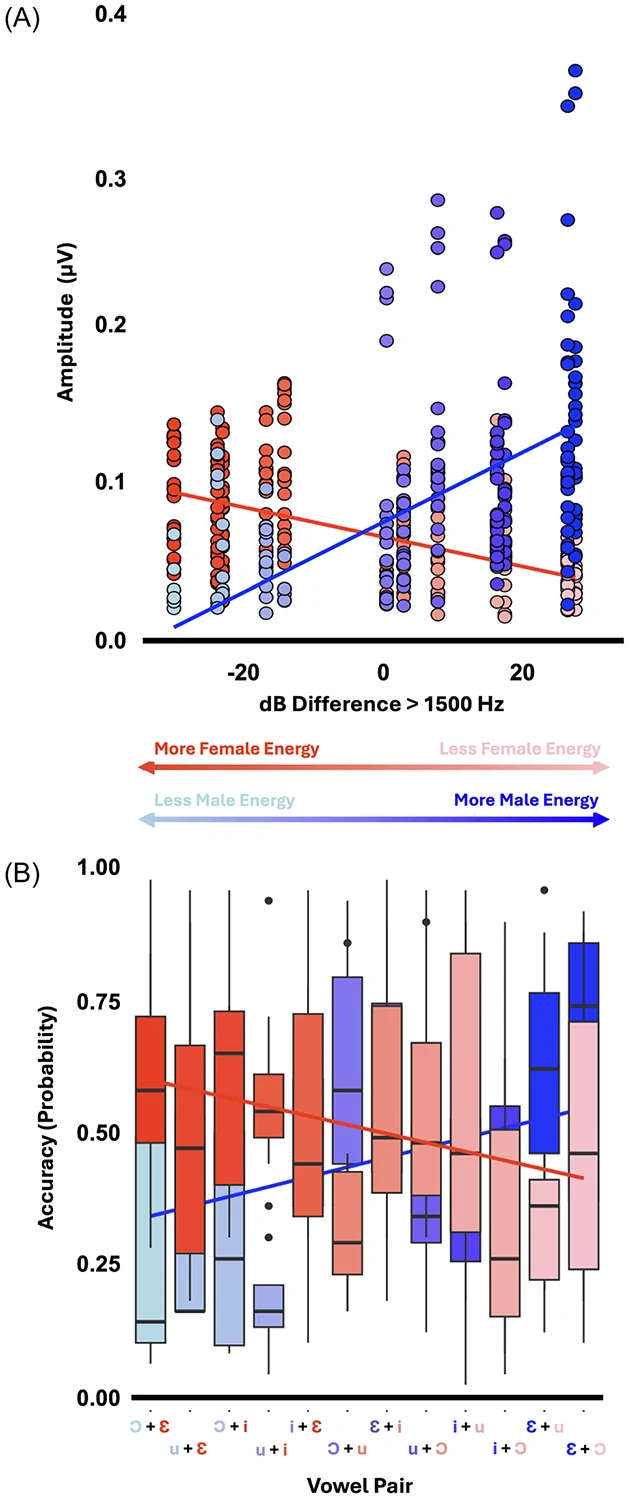
(A) FFR *f*_0_ amplitudes are plotted for male (130 Hz, blue) and female (210 Hz, red) vowels as a function of dB differences > 1500 Hz. The regression trends are plotted using a fitted linear model to smooth the trends for better observations. (B) Box and whisker plots depict vowel identification accuracy for male *f*_0_- and female *f*_0_-cued trials as a function of vowel pair. Note that the vowel pairs are rank-ordered on the *x* axis to correspond with the dB differences in (A) for clearer visual representation. Boxes represent interquartile ranges, where the horizontal line inside the box indicates the median value. The whiskers extend from the box to show the range within 1.5 times the interquartile ranges from the first and third quartiles, capturing the variability outside the center. Outliers are marked by individual points. The regression trends are plotted using a fitted linear model to smooth the trends for better observations.

Relationships between concurrent vowel spectral differences > 1500 Hz and FFR *f*_0_ amplitudes are depicted in Fig. 4(A). This relationship was strong (standardized beta coefficient = 0.6), significant, and positive at 130 Hz {$R_{\text{conditional}}^{2}=0.69$, $R_{\text{marginal}}^{2}=0.27$, *β* = 0.002, 95% CI [0.002,0.003], *p* < 0.001}. Conversely, this relationship was strong (standardized beta coefficient = −0.55), significant, and negative at 210Hz {$R_{\text{conditional}}^{2}=0.58$, $R_{\text{marginal}}^{2}=0.25$, *β* = −0.001, 95% CI [−0.001, −0.0008], *p* < 0.001}. The interaction between dB difference and speaker cue was non-sgnificant for both at 1300 Hz {*β* = −0.001, 95% CI [−0.001, 0.0002], *p* = 0.18} and 210Hz {*β* = 0.0001, 95% CI [−0.0002, 0.0004], *p* = 0.53}. These findings suggest that FFR *f*_0_ amplitudes for each vowel reflect the amount of relative stimulus spectral energy in the mid- to high-frequency range: when male voice mid- to high-frequency harmonics are spectrally dominant, FFR *f*_0_ amplitudes at 130 Hz are large, whereas FFR *f*_0_ amplitudes at 210 Hz are small and vice versa.

#### 2. Relationships between concurrent vowel spectral differences > 1500 Hz and vowel identification accuracy

Relationships between concurrent vowel spectral differences > 1500 Hz and vowel identification accuracy are depicted in Fig. 4(B). The effect of spectral differences > 1500 Hz on male speaker accuracy was moderate (standardized beta coefficient = 0.25), significant, and positive ($R_{\text{conditional}}^{2}=0.48$, $R_{\text{marginal}}^{2}=0.06$, *β* = 0.004, *p* < 0.001). The effect of spectral differences > 1500 Hz on female speaker accuracy was moderate (standardized beta coefficient = −0.27), significant, and negative ($R_{\text{conditional}}^{2}=0.59$, $R_{\text{marginal}}^{2}=0.07$, *b* = −0.004, *p* < 0.001). The interaction between spectral differences > 1500 Hz and speaker cue was significant and negative on accuracy {*β* = −0.008, 95% CI [−0.009, −0.006], *p* < 0.001}, suggesting that spectral differences >1500 Hz and speaker cues independently contribute to the prediction of accuracy. Overall, these results suggest that cued vowel identification accuracy is predicted by the relative amplitude differences of mid- to high-frequency spectral cues in a vowel pair; however, the conditional *R*^2^ values indicate that much of the variance explained by the mixed-effects model is caused by random effects.

#### 3. Mediation analysis

The purpose of mediation analysis was to determine whether the predictive relationships between spectral differences > 1500 Hz (*X*) and identification accuracy (*Y*) were mediated by neural encoding strength of *f*_0_s (i.e., FFR *f*_0_ amplitudes, *M*). Separate mediation analyses were run for male and female FFR *f*_0_ amplitudes and identification accuracies. The mediation analyses used two linear mixed-effects models (LMMs) to account for the random effects of individual subjects such that

Mediatormodel:M∼X+(1∣subject),Outcomemodel:Y∼X+M+X∗M+(1∣subject).

The mediator model and outcome model aimed to quantify the extent to which dB differences > 1500 Hz predicted 130 and 210 Hz FFR amplitude while accounting for individual variability. These models estimated the direct effect of dB difference on accuracy, the indirect effect mediated by FFR amplitude, and their combined total effect. The indirect effect (*α* * *b*) is calculated with the product of the regression coefficient (*α*) for the effect of *X* on *M* from the mediator model and the regression coefficient (*b*) of *M* on *Y* when controlling for *X* and the interaction of *M* and *X* from the outcome model. The direct effect (*c*′) is extracted from the coefficient for *X* on *Y* when controlling for *M* and the interaction of *M* and *X* from the outcome model. Finally, the total effect (*c*) is the sum of indirect and direct effects. The proportion of the total effect mediated is calculated as {(*α* * *b*)/*c*}. By incorporating random effects, these models captured subject-specific variability, ensuring robust estimates of the fixed effects while accounting for within-subject dependencies. The $R_{\text{marginal}}^{2}$, indicating the variance in the dependent variable, is explained by fixed effects (i.e., dB difference > 1500 Hz, FFR amplitude, and their interaction), and the $R_{\text{conditional}}^{2}$, indicating the variance in the dependent variable, is explained by the combined effects of the fixed effect and random effect (i.e., subject).

Whereas the mediator model shows that dB difference explains a notable proportion of the variance in 130 Hz FFR amplitude ($R_{\text{marginal}}^{2}=0.279$), the outcome model indicates that 130 Hz FFR amplitude does not significantly mediate the relationship between dB difference and accuracy. The negligible indirect effect (*α* * *b* = −0.0000184) and the near-zero proportion mediated effect confirms that 130 Hz FFR amplitude contributes minimally to the total effect of dB differences > 1500 Hz on accuracy. Instead, the relationship between dB difference and accuracy is almost entirely direct (*c*′ = 0.0054). Thus, the male vowel mediation analysis demonstrates that the relationship between dB difference and accuracy is primarily direct with no substantial contribution from 130 Hz FFR amplitude as a mediator. The female vowel mediation analysis indicates that 210 Hz FFR amplitude explains a small proportion (24.5%) of the effect of dB difference on accuracy. The remaining 75.5% of the effect is attributed to the direct relationship between dB difference and accuracy. Although the indirect effect is small (*β* = −0.0012), the direct effect remains the dominant pathway (*β* = −0.0036). This analysis suggests that 210 Hz FFR amplitude (*M*) partially mediates the relationship between dB difference (*X*) and accuracy (*Y*), accounting for 24.5% of the total effect. However, the direct pathway remains the primary mechanism driving the relationship with the fixed effects explaining a relatively small proportion of the variance compared to the random effects. Collectively, these results suggest that identification accuracy for female vowels is only partially mediated by neural processes captured by the FFR, whereas no such mediation occurs for male vowels. In both cases, the direct predictive relationship between stimulus spectral differences > 1500 Hz and identification accuracy accounts for the majority of statistical variance.

## IV. DISCUSSION

Perceptual research on concurrent vowel processing has demonstrated the importance of *f*_0_ differences in auditory stream segregation. Although a variety of computational models have been introduced to explain concurrent vowel perception, the neurophysiologic processes that extract *f*_0_ cues from such stimuli are still debated (Assmann and Summerfield, 1989, 1990; Chintanpalli and Heinz, 2013; Culling and Darwin, 1993a,b; de Cheveigné, 2023; Meddis and Hewitt, 1992; Smith et al., 2018). The present study explored *f*_0_-based neural indices of concurrent vowel segregation measured from the human scalp. We simultaneously measured FFRs to 12 concurrent vowel stimuli during a cued vowel identification task and explored parametric relationships between stimulus features (i.e., vowel identity, vowel pairing, and mid- to high-frequency acoustic differences between vowel pairs) and FFR *f*_0_ amplitudes. Further, we explored how mid- to high-frequency acoustic differences impacted cued vowel identification performance and whether these relationships were mediated by subcortical neural channel selection of the concurrent vowel pair *f*_0_s. Whereas the perceptual paradigm in the present study differed from traditional concurrent vowel tasks (e.g., one cued target vowel vs free recall of both vowels) and was more challenging to complete based on average perceptual accuracies [Fig. 4(B)], this study offers new insight into neural processing of concurrent vowel stimuli and may support the use of FFRs in future research on this topic.

FFR *f*_0_ amplitudes were generally smaller for the higher *f*_0_ (210 Hz) compared to the lower *f*_0_ (130 Hz), which replicated previous literature and reflects reduced neural phase locking at higher frequencies (e.g., Bidelman and Powers, 2018). Although every vowel token was calibrated to have equal loudness, FFR *f*_0_ amplitudes to the *same* vowel often differed based on the vowel’s counterpart in the concurrent mixture. This finding suggests that *f*_0_-*based* neural channel selection is more salient in some conditions than others. Notably, the dominant periodicities in the *full-band* concurrent vowel stimulus waveforms (depicted in Fig. 1 autocorrelation plots) were not representative of the observed dominant FFR *f*_0_ frequencies that were depicted in Fig. 3. This finding differs from previous work using single vowels in which FFR autocorrelation functions closely mimic those of the stimuli that evoked them (e.g., Krishnan et al., 2004). In contrast to single vowel data, FFR *f*_0_ amplitudes were predicted by the relative mixture of mid- and high-frequency concurrent vowel harmonics. This is likely because of the significant contributions of unresolved harmonics to FFR *f*_0_ amplitude (de Boer et al., 2020; D’Onofrio et al., 2019; D’Onofrio et al., 2020; Easwar et al., 2015; Easwar et al., 2018; Kessler et al., 2020; Laroche et al., 2013; Xu et al., 2023; Zhu et al., 2013): dominant unresolved mid- to high-frequency harmonics interact to create robust output envelopes that oscillate at the dominant vowel periodicity. Indeed, when the > 1500 Hz filtered stimulus and FFR waveform autocorrelation functions are overlaid (Fig. 3), the dominant periodicities are closely matched for nearly every condition. These results generally align with place-time computational models that segregate vowels based on pooled periodicities of auditory filters along the tonotopic axis (Assmann and Summerfield, 1989, 1990; Meddis and Hewitt, 1992). However, previous perceptual work has mostly pointed to resolved harmonics around F1 as being more important than higher frequencies for concurrent vowel segregation based on *f*_0_ cues (e.g., Culling and Darwin, 1993). This discrepancy may be related to the smaller size of *f*_0_ differences used in previous studies to test pitch-based vowel segregation. When *f*_0_ differences are small (≤1 semitone), identification accuracy of concurrent vowels is driven by resolved harmonics in the *F*1 range; when *f*_0_ differences are large (>4 semitones), unresolved harmonics are weighted more heavily and may support across-formant groupings based on *f*_0_ (Culling and Darwin, 1993a,b; Darwin, 1992; Gardner et al., 1989). In our stimuli, the two voices were very far apart in pitch (130 vs 210 Hz, ~8 semitones) such that unresolved high-frequency harmonics likely provided a clearer contrast between vowels. Thus, the data in the present experiment may reflect neural and perceptual strategies focusing on higher frequency regions of the concurrent vowel mixtures with large *f*_0_ differences that are not used for smaller *f*_0_ differences.

Although there was a strong relationship between spectral differences > 1500 Hz and FFR *f*_0_ strength overall, an exception to this pattern is for the /Ɔ + u/ condition, in which the dominant FFR periodicity was neither at 130 nor 210 Hz, but at ~80 Hz. This result may represent harmonic interactions (e.g., female *f*_0_ – male *f*_0_ or 210 – 130 Hz = 80 Hz) between vowels, as harmonic energy is highly similar across the spectrum. Specifically, the formant structures between vowels nearly overlap, and spectral differences > 1500 Hz are only −2.6 dB. Interestingly, vowel identification accuracies for each vowel in the /Ɔ + u/ pair also differ somewhat from the overall patterns observed in other concurrent vowel combinations and the accuracies predicted by the model (bottom panel, Fig. 3). This may reflect a different neural processing strategy for the /Ɔ + u/ stimulus that focuses on “beating” between competing vowel harmonics as opposed to *f*_0_ differences (e.g., Culling and Darwin, 1993).

The observed relationships between vowel spectral differences > 1500 Hz as well as FFR *f*_0_ amplitudes and vowel identification accuracy indicate that neural *f*_0_ channel selection and target vowel perceptual accuracy were mainly stimulus-driven: vowels with greater mid- to high-frequency energy dominated the FFR, were more perceptually salient, and more likely to be correctly identified. This finding may be a neurophysiologic analogue to previous perceptual data, indicating that there is often a dominant vowel in a concurrent mixture (Chintanpalli and Heinz, 2013; McKeown, 1992; Yellamsetty and Bidelman, 2019). However, the relationship between spectral differences > 1500 Hz and perceptual accuracy was only partially mediated by neural activity represented in the FFR for female target vowels. There was no FFR mediation effect for male target vowels. Notably, four to five participants had FFR *f*_0_ amplitudes at 130 Hz that were larger than the other participants for male-dominated concurrent vowels [see Fig. 4(B)]. Recent work by Bidelman et al. (2024) suggest that abnormally large FFR amplitudes may reflect FFR contamination by periodic contractions of the post-auricular muscle (PAM) at the stimulus *f*_0_. Whereas we expect that PAM contamination would impact all stimuli as a result of their equivalent sensation levels, we cannot rule out this possibility as the reference electrodes were positioned at *M*1 and *M*2. It is possible that an auditory brainstem mediation effect was masked by PAM contamination for male targets. It is also plausible that information contained in the FFR may capture some aspects of neural processing involved in segregation but may not be a direct representation of this process *per se* (e.g., Gockel et al., 2011; Gockel, 2020). For example, concurrent vowel perception may rely more heavily on resolved harmonics in addition to smaller contributions from unresolved harmonics, whereas the FFR is dominated by phase locking to unresolved harmonics. Using concurrent vowel stimuli with random-phase harmonics in the mid- to high-frequencies may help disentangle contributions from unresolved frequencies to perception and neural processing. Interestingly, there were multiple concurrent vowel pairs for which one FFR *f*_0_ component was exceedingly small. This observation is evocative of “harmonic cancellation” in which neural delay lines are implemented to remove one vowel periodicity from a concurrent vowel mixture to reveal the other periodicity (de Cheveigné, 2023). However, our data trend in the opposite direction from what a harmonic cancellation model would predict, as the dominant periodicity > 1500 Hz in the stimulus evokes the dominant periodicity in the FFR.

We observed neither target enhancement nor nontarget suppression of FFRs to cued concurrent vowels. The absence of a brainstem attention modulation effect is consistent with some previous studies (e.g., Varghese et al., 2015) and contradictory to others (e.g., Price and Bidelman, 2021; Cheng et al., 2021; Smith and Cone, 2015). Our observations may support the hypothesis that brainstem auditory processing is driven by exogenous stimulus factors with little top-down influence. Conversely, they may indicate that the measurable effects of transient attention were obscured by our stimulus paradigm, which delivered 20 concurrent vowels pairs in quick succession.

Although our results offer insight into neurophysiologic concurrent vowel processing, several caveats are important to note. First, the paradigm used in the present experiment differs from previous concurrent vowel investigations, making direct comparisons with previous literature difficult. Listeners in this study were asked to focus on a target speaker based on a click train *f*_0_ cue and ignore the other speaker instead of reporting both vowels. This task was more challenging than a “free recall” paradigm, despite the larger *f*_0_ difference between concurrent vowels. Consequently, there was a large range of perceptual accuracy across listeners and concurrent vowel pairs. Additionally, the clustered stimulus paradigm presented 20 concurrent vowel tokens in rapid succession before listeners were able to respond. Whereas this approach expedited FFR recording with minimal neural adaptation and provided listeners with multiple auditory glimpses of the stimulus, it may have been more challenging to hear out the target vowel in this paradigm than a paradigm using a single, longer concurrent vowel mixture per trial. Future investigations employing the FFR to study concurrent vowel processing will use more similar stimuli and paradigms to previous literature to better connect computational modeling, human neurophysiology, and perception to understand how *f*_0_ cues aid speech segregation and streaming.

## Data Availability

The data that support the findings of this study are available from the corresponding author upon reasonable request.
